# Supplementary material for: Low-Dose Tacrolimus Promotes the Migration and Invasion and Nitric Oxide Production in the Human-Derived First Trimester Extravillous Trophoblast Cells In Vitro
Source: Int J Mol Sci. 2022 Jul 29;23(15):8426. doi: 10.3390/ijms23158426 (PMC9369346; doi:10.3390/ijms23158426)

Supplementary Tables for:

# Low-dose Tacrolimus Promotes the Migration and Invasion and Nitric Oxide Production in the Human-derived First Trimester Extravillous Trophoblast Cells *In Vitro*

**By:**
Ahmad J.H. Albaghdadi, Kassandra Coyle, and Frederick W. K. Kan[1]

Department of Biomedical and Molecular Sciences, Faculty of Health Sciences, Queen's University, Kingston, Ontario, Canada K7L 3N6

[1]Corresponding author: Dr. Frederick W. K. Kan

Address: Department of Biomedical and Molecular Sciences, Faculty of Health Sciences, Queen's University, Kingston, Ontario, Canada, K7L3N6

Email: kanfwk@queensu.ca

Telephone: +1 613-533-2863

Supplementary Table S1: Primary antibodies used in this study.

| Antibody ID | Antibody Name | Target Antigen | Clonality | Host Organism | Dilution | Vendor | Catalogue Number | Specific Use |
|---|---|---|---|---|---|---|---|---|
| AB_2166687 | Mouse Anti-PR Monoclonal Antibody, Unconjugated, Clone F-4 | PGR human, mouse, rat | Monoclonal | Mouse | 1:100 | Santa Cruz Biotechnology | SC-166169 | Western blot (WB) detection of the human PGR |
| AB_2864799 | Mouse Anti-PR- Alexa Fluor® 647 Monoclonal Antibody, Conjugated, Clone F-4 | PGR human, mouse, rat | Monoclonal | Mouse | 1:100 | Santa Cruz Biotechnology | sc-166169 AF647 | Immunofluorescent (IF) detection of the human PGR |
| AB_325316 | Phospho-Progesterone Receptor (Ser294) Monoclonal Antibody (608) | Phospho-Progesterone Receptor (Ser294) human | Monoclonal | Mouse | 1:200 | Thermo Fisher Scientific | MA1-414 | Western blot (WB) detection of the human phosphorylated PGR (pPGR) |
| AB_628293 | Stat3 (F-2) antibody | Recombinant protein corresponding to AA 50-240 mapping to amino acids of human origin human, mouse, rat | Monoclonal | Mouse | 1:300 | Santa Cruz Biotechnology | SC-8019 | Western blot (WB) detection of the human STAT3 |
| AB_2491009 | Phospho-Stat3 (Tyr705) (D3A7) XP Rabbit mAb antibody | Phospho-Stat3 (Tyr705) human, mouse, rat | Monoclonal | Rabbit | 1:200 | Cell Signaling Technology | 9145 | Western blot (WB) detection of the human phosphorylated STAT3 (pSTAT3) |

Supplementary Table S1: Primary antibodies used in this study (continued).

| Antibody ID | Antibody Name | Target Antigen | Clonality | Host Organism | Dilution | Vendor | Catalogue Number | Specific Use |
|---|---|---|---|---|---|---|---|---|
| AB_2533121 | Anti-eNOS Monoclonal Antibody, Unconjugated, Clone eNOS-9D10 | eNOS bovine | Monoclonal | Mouse | 1:300 | ThermoFisher Scientific | 33-4600 | Western blot (WB) detection of the human eNOS |
| AB_2533285 | Phospho-eNOS (Ser1179) Polyclonal Antibody | Phospho-eNOS (Ser1179) bovine | Polyclonal | Rabbit | 1:200 | ThermoFisher Scientific | 36-9100 | Western blot (WB) detection of the human phosphorylated $eNOS^{Ser1179}$ ($p\text{-}eNOS^{Ser1179}$) |
| AB_10707645 | Phospho-NOS3 (p-NOS3) (pT495.33) antibody | p-NOS3 (pT495.33) human, mouse, rat | Monoclonal | Mouse | 1:200 | Santa Cruz Biotechnology | SC-136519 | Western blot (WB) detection of the human phosphorylated $eNOS^{Thr495}$ ($p\text{-}eNOS^{Thr495}$) |
| AB_2293930 | FKBP52 Affinity Purified Polyclonal antibody Human/Mouse/Rat | FKBP52 human, mouse, rat | Polyclonal | Goat | 1:200 | R and D Systems | AF4095 | WB and IF detections of the human FKBP52 in |
| AB_626658 | Akt1 (B-1) antibody | AKT1 human, mouse, rat | Monoclonal | Mouse | 1:200 | Santa Cruz Biotechnology | sc-5298 | Wb detection of the protein kinase Akt in HTR-8/SVneo cells |
| AB_2861344 | p-Akt1/2/3 Antibody (C-11) | p-Akt1/2/3 human, mouse, rat | Monoclonal | Mouse | 1:200 | Santa Cruz Biotechnology | sc-514032 | Wb detection of the phosphorylated protein kinase Akt in HTR-8/SVneo cells |

Supplementary Table S2: Secondary antibodies used in this study

| Antibody ID | Antibody Name | Target Antigen | Clonality | Host Organism | Dilution | Vendor | Catalogue Number | Specific Use |
|---|---|---|---|---|---|---|---|---|
| AB_628489 | Mouse anti-goat IgG-FITC | IgG goat | Monoclonal | Mouse | 1:3000 | Santa Cruz Biotechnology | 33-4600 | Secondary Ab used in the IF detection of the human FKBP52 |
| AB_631746 | Goat anti-rabbit IgG-HRP antibody | IgG rabbit | Polyclonal | Rabbit | 1:5000 | Santa Cruz Biotechnology | SC-2004 | Secondary Ab used in WB |
| AB_2536527 | Goat anti-Mouse IgG (H+L) Cross-Adsorbed Secondary Antibody, HRP | IgG (H+L) mouse | Polyclonal | Goat | 1:1000 | ThermoFisher Scientific | G21040 | Secondary Ab used in WB |

Supplementary Figures for:

# Low-dose Tacrolimus Promotes the Migration and Invasion and Nitric Oxide Production in the Human-derived First Trimester Extravillous Trophoblast Cells *In Vitro*

**By:**

Ahmad J.H. Albaghdadi, Kassandra Coyle and Frederick W. K. Kan[1]

Department of Biomedical and Molecular Sciences, Faculty of Health Sciences, Queen's University, Kingston, Ontario, Canada K7L 3N6

[1]Corresponding author: Dr. Frederick W. K. Kan

Address: Department of Biomedical and Molecular Sciences, Faculty of Health Sciences, Queen's University, Kingston, Ontario, Canada, K7L3N6

Email: kanfwk@queensu.ca

Telephone: +1 613 533-2863

**Supplementary Figure Legends**

**Supplementary Figure S1**:

Detection of PGR mRNA in HTR8/SVneo cells treated with DMSO only (Control), tacrolimus (TAC), L-NAME, TAC+L-NAME, Mifepristone and TAC+ Mifepristone, respectively. Tacrolimus significantly ($p < 0.05$) induced the expression of the PGR mRNA in the HTR-8/SVneo cells irrespective of the presence or absence of the eNOS and PGR inhibitors; L-NAME and Mifepristone, respectively. mRNA copies were calculated using the comparative threshold cycle (ΔΔCT) method with normalization to GAPDH. *R* values were measured as the negative values of ΔΔCT as exponent of 2 according to the equation: R = R = 2ˆ (−ΔΔCr) where ΔΔCT = ΔCT (Target) − ΔCT (Endogenous Control). GAPDH primers were utilized as positive controls. Negative controls without RNA and without reverse transcriptase were also performed.

**Supplementary Figure S2:**

Effects of low-dose tacrolimus on IL6 secretion in HTR8/SVneo cells. A highly sensitive human IL6 ELISA kit (Catalog number: EH2IL6, Invitrogen) was used to detect alterations in concentrations of this cytokine in HTR8/SVneo cells in conditioned media after 24 hours of treatment with low-dose tacrolimus (10ng/ml) alone or in a combination formulation with L-NAME or mifepristone. Compared to the DMSO-free control, a significant trend ($p = 0.06$) increase in IL6 production was observed among all treatment conditions. However, compared to DMSO-only treated controls, none of the treatment conditions used in this study was able to influence the release of IL6 in HTR8/SVneo cells. Data are presented as mean ± SD.

**Supplementary Figure S3:**

Effects of low-dose tacrolimus on TNFα secretion in HTR8/SVneo cells. A highly sensitive human TNFα ELISA kit (Catalog number: BMS223HS, Invitrogen) was used to detect alterations in concentrations of this cytokine in HTR8/SVneo cells in conditioned media after 24 hours of treatment with low-dose tacrolimus (10ng/ml) alone or in a combination formulation with L-NAME or mifepristone. Compared to DMSO-only treated controls, Low-dose tacrolimus and L-NAME significantly increased the concentrations of TNFα in the condition medium of HTR8/SVneo cells. Data are presented as mean ± SD.

**Supplementary Figure S4:**

A: Representative Western blot detection of protein expression and phosphorylation of Akt in HTR8/SVneo cells treated with DMSO-only (Control: Ctrl), tacrolimus (TAC), mifepristone (Mife), and TAC+ mifepristone (TAC+ Mife), respectively. B: Histogram analysis of the relative expression of pAkt/Akt in HTR8/SVneo cells demonstrating a significant time-dependent increase in protein expression and phosphorylation of Akt in the TAC- and TAC+ Mife treated cells over 72 hours of incubation. MWM: Molecular Weight Markers.

**Supplementary Figure S1**

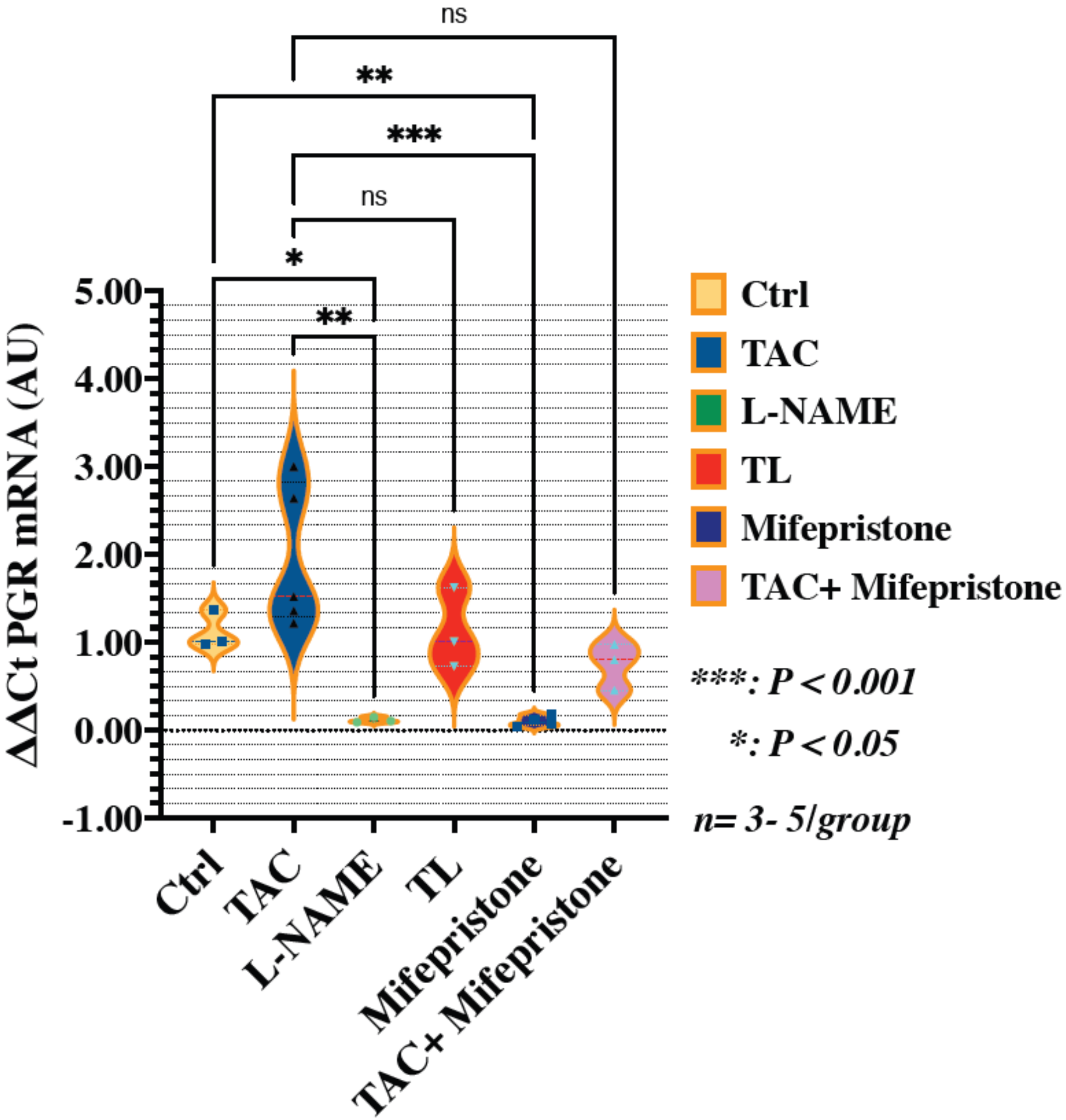

**Supplementary Figure S2**

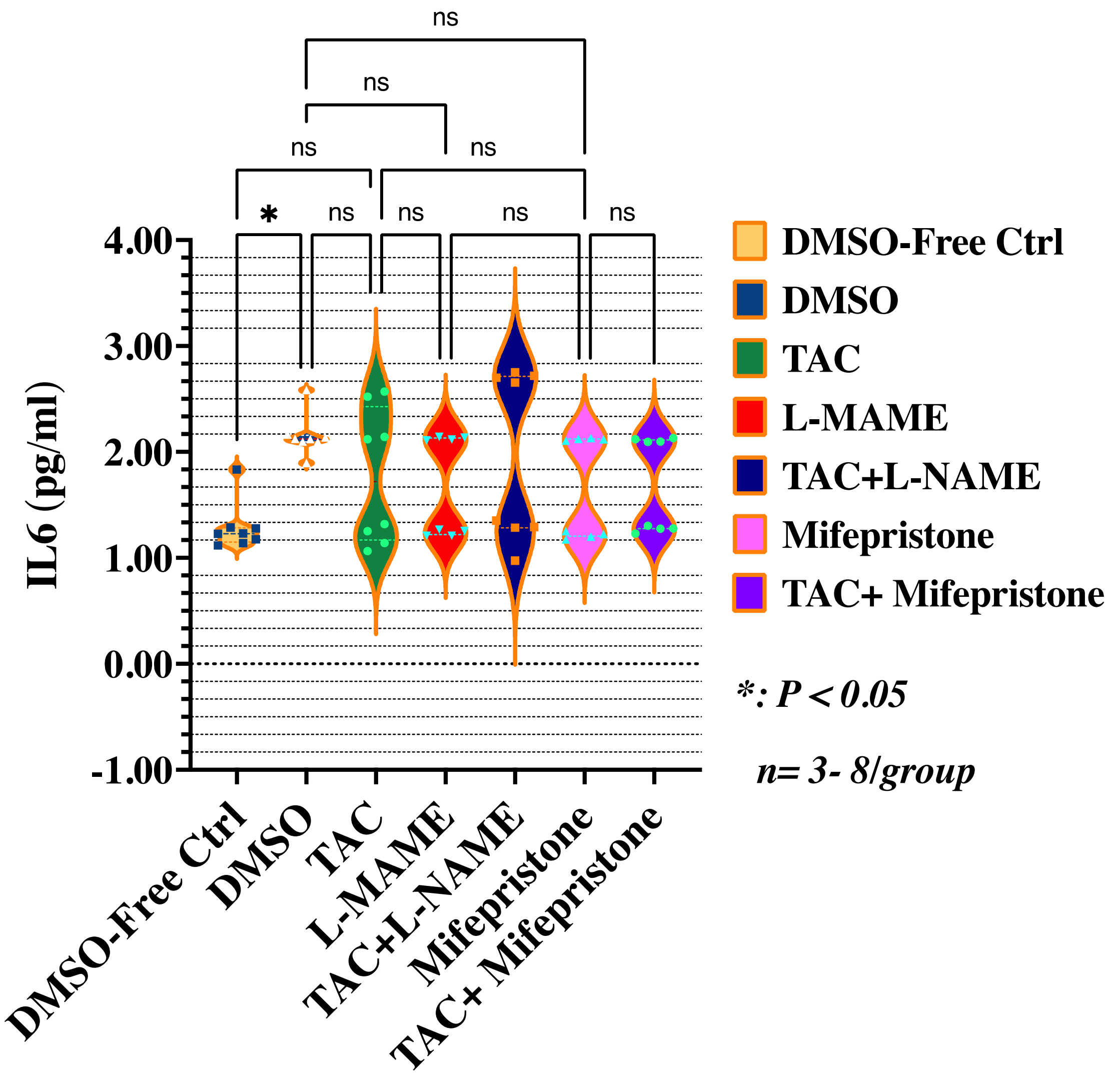

**Supplementary Figure S3**

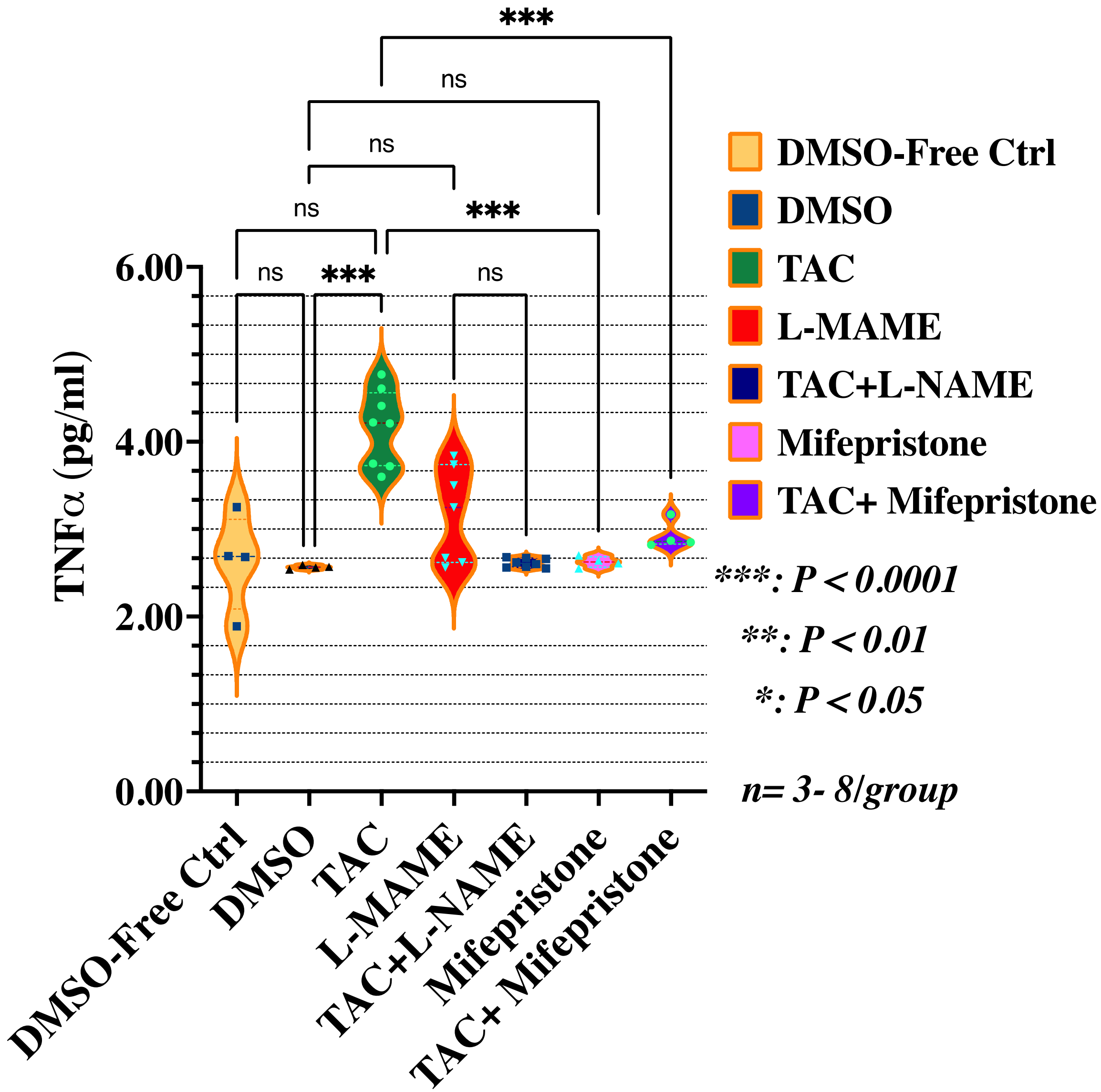

**Supplemental Figure S4**

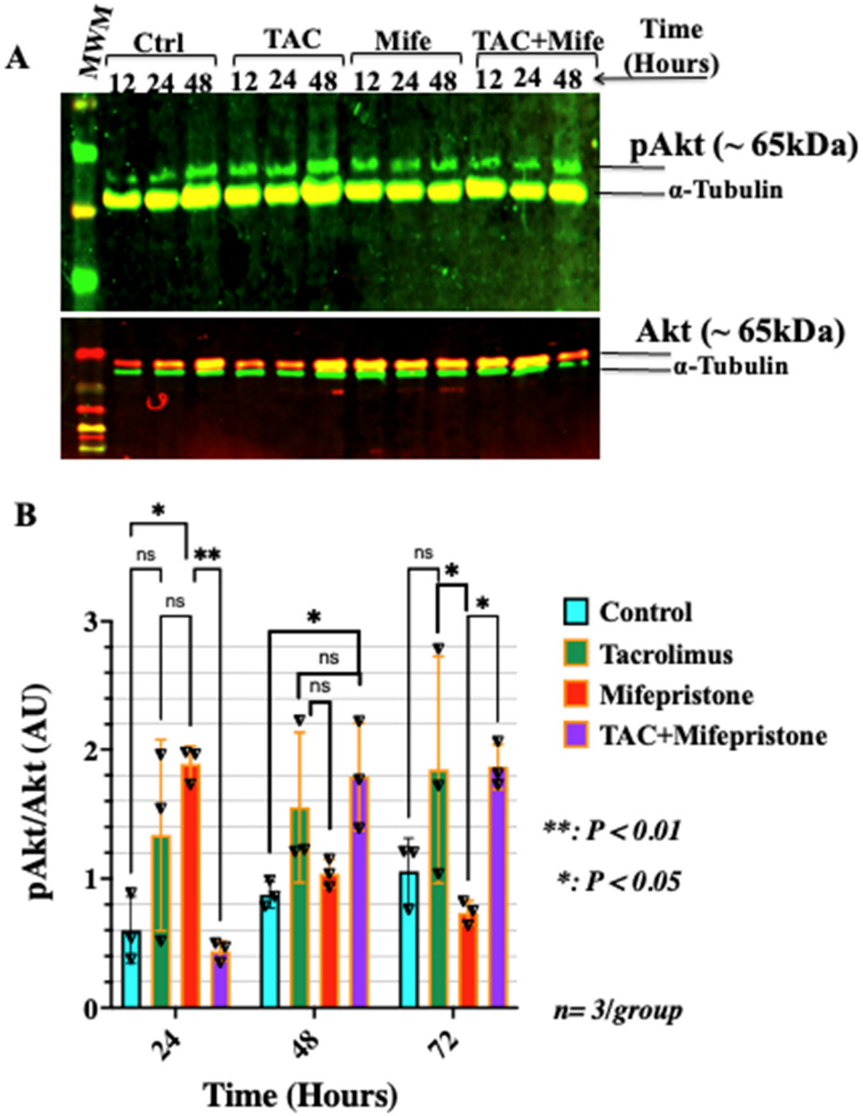

Supplement: Supplementary file 1 [file ijms-23-08426-s001.zip › ijms-1363700-Supplementary.pdf]
